# Association of erectile dysfunction with tinnitus: a nationwide population-based study

**DOI:** 10.1038/s41598-021-86441-6

**Published:** 2021-03-26

**Authors:** Yen-Fu Cheng, Sudha Xirasagar, Nai-Wen Kuo, Shiu-Dong Chung, Herng-Ching Lin

**Affiliations:** 1 Department of Medical Research, Taipei Veterans General Hospital, Taipei, Taiwan; 2 Department of Otolaryngology-Head and Neck Surgery, Taipei Veterans General Hospital, Taipei, Taiwan; 3 Faculty of Medicine, National Yang Ming Chiao Tung University, Taipei, Taiwan; 4 Institute of Brain Science, National Yang Ming Chiao Tung University, Taipei, Taiwan; 5 Research Center of Sleep Medicine, College of Medicine, Taipei Medical University, Taipei, Taiwan; 6 Department of Health Services Policy and Management, Arnold School of Public Health, University of South Carolina, Columbia, USA; 7 Department of Health Care Administration, Taipei Medical University, Taipei, Taiwan; 8 Division of Urology, Department of Surgery, Far Eastern Memorial Hospital, Ban Ciao, Taipei, Taiwan; 9 Sleep Research Center, Taipei Medical University Hospital, Taipei, Taiwan; 10 School of Health Care Administration, Taipei Medical University, 250 Wu-Hsing St, Taipei, 110 Taiwan

**Keywords:** Neurology, Risk factors, Urology

## Abstract

With many previous studies indicating a higher prevalence of sexual problems in patients with tinnitus, the association between tinnitus and erectile dysfunction (ED) has become an interesting topic that warrants further research. In our study, we hypothesized that tinnitus may be associated with ED and aimed to further explore the relationship between these two medical conditions using a nationwide population-based database. After retrieving data of 19,329 patients with ED and 19,329 propensity score-matched patients without ED (controls) from Taiwan’s National Health Insurance Dataset, we defined the diagnosis date (the date of the first ED claim) for patients with ED as the index date for cases, and the date of the first utilization of ambulatory care by patients without ED during the index year of their matched case as the index date for controls. We found that 1247 out of 38,658 sampled patients (3.23%) had received a tinnitus diagnosis within the year before the index date, with 792 (4.10%) from cases and 455 (2.35%) from controls. We then utilized multiple logistic regression analysis and observed that cases were more likely to have had a prior tinnitus diagnosis compared to controls (OR 1.772; 95% CI 1.577–1.992; *p* < 0.001). Lastly, we adjusted the data for co-morbid medical disorders and social economic factors, with the end results showing that cases were more likely than controls to have a prior diagnosis of tinnitus (OR 1.779, 95% CI 1.582–2.001, *p* < 0.001). Through our investigation, we have ultimately detected a novel association between ED and tinnitus and urge physicians to be alert to the possibility of the development of ED in patients treated for tinnitus.

## Introduction

Erectile dysfunction (ED) is a type of sexual dysfunction characterized by the inability to achieve or maintain an erection that is sufficient for satisfactory sexual performance. It is a common condition that primarily affects men older than 40 years, with negative impacts on interpersonal relationship, mood, self-esteem and quality of life. ED affects up to half of men during their lives, and epidemiological studies have reported prevalence rates ranging from 17–52% among men aged over 40 years^1–3^. ED is psychosomatic condition with individually varying degrees of medical/somatic and psychological contributors: while vasculogenic, neurogenic, endocrine factors can be identified in many cases, psychological factors such as anxiety, low mood, or interpersonal difficulties can likewise lead or contribute to the onset or maintenance of ED^4–6^.

Tinnitus is the perception of a phantom sound in the absence of a corresponding external auditory stimulus. Tinnitus is reported to be prevalent in up to 15% of population globally, and 10% of them suffer quality of life impacts with some patients experiencing severe and debilitating tinnitus causing sleep disturbance, distress, poor concentration, depression and anxiety^7,8^. Although its precise pathophysiology remains unclear, tinnitus is thought to be the result of maladaptive cortical plasticity in response to anatomic and functional changes in the neural networks of the auditory and non-auditory systems involved in hearing^9^. Whilst tinnitus has been traditionally regarded as a symptom resulting from inner ear damage (resulting from noise exposure or ototoxic drugs), recent studies have demonstrated that tinnitus emerges from an intersction of both peripheral and central neurological pathways contributors to which may both involve medical (temporomandibular disorders, encephalitis), endocrinological, medication-related or psychological factors (e.g. vulnerability-stress interactions)^10–19^.

Harrop-Griffiths et al. and Muluk et al. reported a higher prevalence of sexual problems in patients with tinnitus^15,16^. However, to our knowledge, the association between tinnitus and ED has not been studied. Therefore, we hypothesized that tinnitus may be associated with ED. This study aims to explore the relationship between these two medical conditions using a nationwide population-based database.

## Methods

### Database

We retrieved data for this retrospective case–control study from Taiwan’s National Health Insurance Dataset (NHIRD), which consists of curated administrative data from the Taiwan National Health Insurance (NHI) program. The NHIRD consists of claims data and registration files of over 99% of all Taiwanese residents (*n* = 23.72 million). Many researchers in Taiwan have used the NHIRD to perform clinical-epidemiological studies published in internationally peer-reviewed journals. The NHIRD provides unique opportunity to explore the association of ED with prior tinnitus using population-based data.

This study was approved by the Institutional Review Board of Taipei Medical University (TMU-JIRB N202006067). This study adheres to the STROBE guidelines for research reporting standards, and is compliant with the Declaration of Helsinki. This study used the administrative dataset so the informed consent was waived and approved by the Institutional Review Board of Taipei Medical University.

### Selection of cases and controls

We first retrieved 19,337 patients who were aged between 40 ~ 79 years old and had received their first diagnosis of ED ((ICD-9-CM code 607.84, impotence of organic origin) or (ICD-10-CM code N52.9, male erectile dysfunction, unspecified)) during ambulatory care visits between January 2016 and December 2017 from the NHIRD. In Taiwan, physicians make an ED diagnosis based on the results of a self-administered IIEF-5 (International Index of Erectile Dysfunction) questionnaire. We attribute high degree of validity to ED diagnoses made in Taiwan because diagnosed cases are probably the tip of the iceberg due to cultural taboos associated with visiting a physician for sexual problems in Taiwan. To enhance diagnostic validity, we included patients only if they had at least one claim showing an ED diagnosis filed by a urologist, leading a case group sample of 19,329 patients with ED being included in the study. The diagnosis date was the date of the first ED claim for ED, which was the index date for cases.

We select controls out of the remaining NHIRD enrollees. We first excluded patients with a history of ED prior to January 2016, and selected those aged between 40 and 79 years old. Thereafter, we selected propensity score-matched controls, one control per ED case matched to cases by using demographic variables (age, sex, monthly income, geographic location and urbanization level of the patient’s residence) and comorbidities associated with a higher risk for ED, hyperlipidemia, diabetes, coronary heart disease, and hypertension. Identified controls were matched with ED cases based on identifying a claim for any ambulatory medical service in the index year of the case. Furthermore, we defined the date of the control patient’s first utilization of ambulatory care during the index year of their matched case as their index date.

### Exposure assessment

Tinnitus during a one-year period before the index date was the exposure of interest. We identified cases and controls with prior tinnitus based on ICD-9-CM code 388.3 or ICD-10-CM code H93.1 present in at least one claim within the year preceding the index date. In addition, we only included tinnitus cases who had received at least one of tinnitus diagnoses being made by an otorhinolaryngologist.

### Statistical analysis

We carried out statistical analyses using the SAS system (SAS System for Windows, Version 8.2, SAS Institute Inc, Cary, NC). We compared the distributions of cases and controls on demographic variables and medical co-morbidities using chi-square tests. We used logistic regression analysis to examine the association of ED with prior newly-onset tinnitus. We used *p* ≤ 0.05 to assess statistical significance.

## Results

Table 1 shows the descriptive data for 19,329 cases and 19,329 controls. The mean age of the sample 38,658 patients was 57.6 years (standard deviation, SD 9.4), similar in both groups (*p* = 0.982). There were no significant differences between cases and controls on monthly income, geographic location and residential urbanization level, hyperlipidemia, diabetes, hypertension and coronary heart disease.

**Table 1:** Demographic characteristics of patients diagnosed with erectile dysfunction during 2016–2017 and control patients in Taiwan (n = 38,658).

Variable	Patients with erectile dysfunction (*n* = 19,329)		Controls (*n* = 19,329)		P value
	Total No	%	Total No	%	
Age, mean (SD)	57.58	(9.44)	57.58	(9.44)	0.982
**Monthly income**					
< NT$1–15,841	3739	19.34%	3776	19.54%	0.853
NT$15,841–25,000	5903	30.54%	5863	30.33%	
≥ NT$25,001	9687	50.12%	9690	50.13%	
**Geographic region**					
Northern	9102	47.09%	9136	47.27%	0.941
Central	4457	23.06%	4448	23.01%	
Southern	5410	27.99%	5373	27.80%	
Eastern	360	1.86%	372	1.92%	
**Urbanization level**					
1 (most urbanized)	5939	30.73%	5927	30.66%	0.970
2	6015	31.12%	6006	31.07%	
3	3232	16.72%	3232	16.72%	
4	2141	11.08%	2178	11.27%	
5 (least urbanized)	785	4.06%	757	3.92%	
Other	1217	6.30%	1229	6.36%	
Hyperlipidemia	8408	43.50%	8405	43.48%	0.975
Diabetes	5627	29.11%	5630	29.13%	0.973
Hypertension	8854	45.81%	8929	46.19%	0.444
Coronary heart disease	3848	19.91%	3869	20.02%	0.789

The prevalence of prior tinnitus was presented in Table 2. We found that 1247 out of 38,658 sampled patients (3.23%) had received a tinnitus diagnosis within the year before the index date, with 792 (4.10%) from cases and 455 (2.35%) from controls. We then utilized multiple logistic regression analysis and observed that cases were more likely to have had a prior tinnitus diagnosis compared to controls (OR 1.772; 95% CI 1.577–1.992; *p* < 0.001).

**Table 2:** Prevalence of tinnitus and crude odds ratio of prior tinnitus among cases vs. controls.

Presence of prior tinnitus	Total (*n* = 38,658)		Patients with erectile dysfunction (*n* = 19,329)		Controls (*n* = 19,329)	
	*n*, %		*n*, %		*n*, %	
Yes	1247	3.23%	792	4.10%	455	2.35%
No	37,411	96.77%	18,537	95.90%	18,874	97.65%
OR (95% CI)	–		1.772*** (1.577–1.992)		1.00	

*OR* odds ratio.

***Indicates p < 0.001.

Table 3 presents the covariate-adjusted odds ratio (OR) for ED among sample patients. After adjusting for age, monthly income, geographic location, urbanization level, hypertension, diabetes, coronary heart disease, and hyperlipidemia, cases were more likely than controls to have a prior diagnosis of tinnitus (OR 1.779, 95% CI 1.582–2.001, *p* < 0.001).

**Table 3:** Covariate-adjusted odds of prior tinnitus among erectile dysfunction patients vs. controls (n = 38,658).

Variables	Presence of erectile dysfunction		
	Odds ratio	95% CI	P value
Prior tinnitus	1.779	(1.582–2.001)	< 0.001
Age	1.039	(1.032–1.046)	< 0.001
**Monthly income**			
< NT$1–15,841 (ref)			
NT$15,841–25,000	1.093	(0.928–1.288)	0.286
≥ NT$25,001	1.115	(0.955–1.301)	0.170
**Geographic region**			
Northern (ref)			
Central	1.287	(1.105–1.499)	0.001
Southern	1.006	(0.868–1.166)	0.937
Eastern	1.014	(0.659–1.562)	0.948
**Urbanization level**			
1 (most urbanized) (ref)			
2	1.153	(0.991–1.343)	0.066
3	1.03	(0.852–1.246)	0.756
4	1.275	(1.038–1.565)	0.021
5	1.141	(0.840–1.550)	0.398
> 5 (least urbanized)	1.323	(1.040–1.682)	0.022
Hyperlipidemia	1.354	(1.195–1.533)	< 0.001
Diabetes	0.947	(0.832–1.078)	0.409
Hypertension	0.956	(0.844–1.082)	0.473
Coronary heart disease	1.286	(1.125–1.469)	< 0.001

*CI* confidence interval.

Table 4 shows the adjusted odds ratio of prior tinnitus among cases vs. controls stratified by the presence of hypertension, hyperlipidemia, diabetes and coronary heart disease and age group. We found that the association between ED and tinnitus still sustains regardless of the presence hypertension, hyperlipidemia, diabetes and coronary heart disease and age group.

**Table 4:** Adjusted odds ratio of prior tinnitus among cases vs. controls stratified by the presence of hypertension, hyperlipidemia, diabetes, coronary heart disease and age group.

Variable	Patients with erectile dysfunction (*n* = 19,329)	Controls (*n* = 19,329)
	Adjusted OR (95% CI)	
Patients with hypertension	1.863 (1.583–2.193)	1.000
Patients without hypertension	1.683 (1.422–1.933)	1.000
Patients with hyperlipidemia	1.867 (1.587–2.197)	
Patients without hyperlipidemia	1.676 (1.416–1.985)	
Patients with diabetes	1.814 (1.477–2.228)	1.000
Patients without diabetes	1.753 (1.520–2.021)	1.000
Patients with coronary heart disease	1.752 (1.404–2.187)	1.000
Patients without coronary heart disease	1.783 (1.553–2.047)	1.000
Patients aged 40 ~ 64 years	1.688 (1.459–1.954)	1.000
Patients aged 65 ~ 79 years	1.941 (1.594–2.362)	1.000

*OR* odds ratio.

## Discussion

To our knowledge this study is the first to investigate the association between newly-onset tinnitus and the subsequent development of ED a nationwide population-based database. Using a propensity-score matched case–control study approach, we found that the odds are 1.78 times higher than for those without a diagnosis of ED that they also have reported tinnitus within the year before ED onset.

Both tinnitus and ED may be underlined by multifactorial influencing factors, including organic and psychosocial factors. In the organic aspect, sex hormones may play a role in the association between tinnitus and ED. Testosterone, the dominant androgenic hormone, is considered to be the key regulating hormone for penile development and functioning^20,21^. In addition to its role in the maintenance and development of masculine features, testosterone is also involved in erectile function and sex drive^21,22^. Studies have shown a direct involvement of testosterone in cavernous smooth muscle cell functioning through its role in molecular pathways such as the nitric oxide pathway, RHO-associated protein kinase, phosphodiesterase type 5 and the adrenergic response^23–25^. Testosterone and its androgenic metabolite (*dihydrotestosterone*) exert their biological effects directly through binding to the androgen receptors. In addition to penile tissue, androgen receptors are also found in the cochlear nucleus, the first neuronal processor of multisensory stimuli in the central auditory pathway^26–28^. Beyond this finding, however, little is known about the activity of androgen in the auditory system.

Tinnitus has been reported to be caused by dysregulated neural synchrony across neural ensembles along the auditory pathways^29,30^. As the first central site of multisensory integration, the cochlear nucleus receives inputs from the cochlea and peripheral auditory nerve, and projects the integrated input upwards to the auditory cortex through the inferior colliculus and thalamus. Animal studies have shown androgen receptors in the cochlear nucleus of the brainstem and the cochlear apparatus of the peripheral auditory system^26–28^. It is thought that androgen may have a neuroprotective effect against the destructive impacts of noise; less noise-generated damage is observed in both peripheral cochlear hair cells and the neurons of the cochlear nucleus in control male animals than in orchidetomized animals^28^. Androgen receptors were found to be downregulated in the cochlear nucleus of aging animals, along with similar changes in other sensory-related cranial nuclei, suggesting that a decline in functioning androgen receptors may contribute to impaired copulatory behavior in aging animals^27^. Androgen-related changes may thus play a role in the pathogenesis of tinnitus among men, and further research is required to explore the relationship between androgen and tinnitus.

ED has emerged as an important marker for cardiovascular health, and is regarded as a reliable proxy for general health status^2,31,32^. In fact, more than 80% of erectile dysfunction can be attributed to an organic etiology, with vasculogenic etiology being the most common among organic etiologies^20^. Several studies have found that vascular diseases, including cardiovascular diseases, dyslipidemia, peripheral vascular disease, and ischemic heart disease, are direct risk factors for the occurrence of tinnitus^33,34^. Due to the absence of collateral blood supply in the cochlea and its high metabolic requirements, the cochlea is highly susceptible to circulatory compromise arising out of systemic or local causes. The stria vascularis is the major regulator of ionic concentration of the endolymph within the inner ear, and the endothelial cells of the capillaries of the stria vascularis form a critical blood-labyrinth barrier^35,36^. Disease processes that disrupt the cochlear vasculature and stria vascularis cause a breakdown of the blood-labyrinth barrier, potentially leading to symptoms of tinnitus due to altered auditory processing^34^. The association between tinnitus and the subsequent ED may be explained by shared causal mechanisms underlying both the vascular and endothelial dysfunction of tinnitus and the penile function disruption in ED. Hence, tinnitus could precede the occurrence of ED and may be a proxy for both sexual and cardiovascular health.

Apart from an organic pathophysiological pathway, psychological factors are also likely to contribute to the co-occurrence of both tinnitus and ED. for example, low mood is a common epiphenomenon of ED and are overrepresented among patients with tinnitus. Epidemiological studies have shown common (risk) factors to comprise mood difficulties, anxiety and personality vulnerabilities, as well as broader 'stress'-related factors and secondary problems such as sleep difficulties^2,37^. Anxiety, for example, can lead to a disproportionate focus on the quality and quantity of erection and create a cognitive distraction that affects sexual intimacy, sexual arousal and therefore subsequent erection^20,38^. Thus, underlying emotional factors such as low mood, performance anxiety, fear of intimacy, anger, or fear of negative evaluation may aggravate or maintain ED alongside an individual differences continuum that may also affect tinnitus-related distress or symptom maintenance respectively^39^. As both tinnitus and ED are likely to be caused and maintained by interrelating psychobiological contributors, both diagnostic efforts and treatment approaches should consider and conceptualize a multifactorial set of biopsychosocial factors. A thorough psychosomatic understanding of both symptoms as well as their commonalities may be more effective than pharmaceutical management alone and may improve aspects of psychological and physical health beyond the mere index symptoms.

A strength of this study is the use of a nationwide population-based dataset that covers more than 99% of the population. Previous studies of the association between tinnitus and ED were limited by small case numbers, reliance on self-reported data based on interviews and questionnaires, and the absence of a robust comparison group. This study overcomes the limitations of self-report bias by using urologist-diagnosed cases for diagnostic validity, and overcomes selection bias by comparing cases with propensity-score matched controls selected from the entire population. Large case numbers from a nation-wide claims dataset also provides sufficient statistical power to detect differences between cases and controls.

The study has some limitations. First, ED is associated with considerable social and cultural stigma. Discussing sexual dysfunction or sexual performance remains a sensitive issue in Asian countries like Taiwan, causing many men to accept sexual dysfunction as results of aging or fate^40,41^ Thus, affected Taiwanese men are likely reluctant to seek medical help and medical professionals can be sensitized to and valuably contribute to de-stigmatizing this often disabling condition. Unfortunately, also, pharmacological treatment of ED is not covered under Taiwan’s NHI program. These factors may contribute to underreporting of ED among control patients, which should bias our results towards the null hypothesis. Our finding of a significant and large effect size supports the existence of a true association between ED and prior tinnitus. Second, the administrative dataset and ICD-10-CM used in this study did not allow us to distinguish between bothersome and non-bothersome tinnitus, the severity/burden of the tinnitus or objective vs subjective tinnitus. Similarly, self-report measure (IIEF-5) as the ED diagnostic standard may warrant further research on the validity of this questionnaire in clinical research and clinical practice. Third, although this study identified ED cases by ICD-9-CM code 607.84 (impotence of organic origin) or ICD-10-CM code N52.9 (male erectile dysfunction, unspecified), we could not rule out the possibility that the patient sample included both individuals with more 'functional' ED presentations. However, we wish to emphasize that the psychological impact of ED is of course to be considered in patients with both organic and non-organic causes and the need for a comprehensive assessment and treatment plan should always consider the psychosocial impact of the symptom, as applicable. Finally, data on other important factors affecting the likelihood of ED and tinnitus are not available in claims data, such as laboratory data and lifestyle factors such as smoking, alcohol consumption, body mass index and partner status, limiting our ability to account for vasculogenic and endocrine factors. Finally, epidemiological association does not imply biological causation, which requires prospective studies as well as neurobiologic studies to confirm causal associations and identify the pathophysiologic mechanisms underlying tinnitus and ED.

In conclusion, this investigation detected a novel association between ED and tinnitus after adjusting for co-morbid medical disorders and social economic factors. As both tinnitus and ED can be caused and maintained by both somatic (e.g. cardiovascular conditions, diabetes) and psychological factors (low mood, anxiety, relationship problems), physicians should raise awareness of psychosomatic contributors that allow for a humane, holistic and comprehensive multifactorial treatment strategy.

## Data Availability

The National Health Insurance Research Database, which has been transferred to the Health and Welfare Data Science Center (HWDC). Interested researchers can obtain the data through formal application to the HWDC, Department of Statistics, Ministry of Health and Welfare, Taiwan (http://dep.mohw.gov.tw/DOS/np-2497-113.html).

## References

[1] Corona G (2010). Age-related changes in general and sexual health in middle-aged and older men: Results from the European Male Ageing Study (EMAS). J. Sex. Med..

[2] Feldman HA, Goldstein I, Hatzichristou DG, Krane RJ, McKinlay JB (1994). Impotence and its medical and psychosocial correlates: Results of the Massachusetts Male Aging Study. J. Urol..

[3] Corona G, Rastrelli G, Maseroli E, Forti G, Maggi M (2013). Sexual function of the ageing male. Best Pract. Res..

[4] Rosen RC (2001). Psychogenic erectile dysfunction. Classification and management. Urol. Clin. N. Am..

[5] Maseroli E (2015). Prevalence of endocrine and metabolic disorders in subjects with erectile dysfunction: A comparative study. J. Sex. Med..

[6] Allen MS, Walter EE (2019). Erectile dysfunction: An umbrella review of meta-analyses of risk-factors, treatment, and prevalence outcomes. J. Sex. Med..

[7] Bhatt JM, Lin HW, Bhattacharyya N (2016). Prevalence, severity, exposures, and treatment patterns of tinnitus in the United States. JAMA Otolaryngol..

[8] Heller AJ (2003). Classification and epidemiology of tinnitus. Otolaryngol. Clin. North Am..

[9] Shore SE, Roberts LE, Langguth B (2016). Maladaptive plasticity in tinnitus–triggers, mechanisms and treatment. Nat. Rev. Neurol..

[10] Cheng Y-F (2020). Increased risk of tinnitus following a trigeminal neuralgia diagnosis: A one-year follow-up study. J. Headache Pain.

[11] Bousema EJ, Koops EA, van Dijk P, Dijkstra PU (2018). Association between subjective tinnitus and cervical spine or temporomandibular disorders: A systematic review. Trends Hear..

[12] Nondahl DM (2011). Tinnitus and its risk factors in the Beaver Dam offspring study. Int. J. Audiol..

[13] Yu J-N (2019). Association between menstrual cycle irregularity and tinnitus: A nationwide population-based study. Sci. Rep..

[14] Lai JT, Liu CL, Liu TC (2017). Hormone replacement therapy for chronic tinnitus in menopausal women: Our experience with 13 cases. Clin. Otolaryngol..

[15] Park RJ, Moon JD (2014). Prevalence and risk factors of tinnitus: the Korean National Health and Nutrition Examination Survey 2010–2011, a cross-sectional study. Clin. Otolaryngol..

[16] Cheng Y-F (2020). A population-based case-control study of the association between cervical spondylosis and tinnitus. Int. J. Audiol..

[17] Biehl R, Boecking B, Brueggemann P, Grosse R, Mazurek B (2019). Personality traits, perceived stress, and tinnitus-related distress in patients with chronic tinnitus: Support for a vulnerability-stress model. Front. Psychol..

[18] Salviati M (2014). Tinnitus: Clinical experience of the psychosomatic connection. Neuropsychiatr. Dis. Treat..

[19] Boecking B (2020). Tinnitus-related distress and pain perceptions in patients with chronic tinnitus—Do psychological factors constitute a link?. PLoS ONE.

[20] Yafi FA (2016). Erectile dysfunction. Nat. Rev. Dis. Primers..

[21] Isidori AM (2014). A critical analysis of the role of testosterone in erectile function: From pathophysiology to treatment-a systematic review. Eur. Urol..

[22] Corona G (2017). Meta-analysis of results of testosterone therapy on sexual function based on international index of erectile function scores. Eur. Urol..

[23] Hull EM (1999). Hormone-neurotransmitter interactions in the control of sexual behavior. Behav. Brain Res..

[24] Sopko NA, Hannan JL, Bivalacqua TJ (2014). Understanding and targeting the Rho kinase pathway in erectile dysfunction. Nat. Rev. Urol..

[25] Zhang X-H (2005). Testosterone regulates PDE5 expression and in vivo responsiveness to tadalafil in rat corpus cavernosum. Eur. Urol..

[26] Smeti I, Assou S, Savary E, Masmoudi S, Zine A (2012). Transcriptomic analysis of the developing and adult mouse cochlear sensory epithelia. PLoS ONE.

[27] McGinnis MY, Yu WH (1995). Age-related changes in androgen receptor levels in cranial nerve nuclei of male rats. Brain Res. Bull..

[28] Willott JF (2009). Effects of sex, gonadal hormones, and augmented acoustic environments on sensorineural hearing loss and the central auditory system: Insights from research on C57BL/6J mice. Hear. Res..

[29] Heeringa AN, Wu C, Shore SE (2018). Multisensory integration enhances temporal coding in ventral cochlear nucleus bushy cells. J. Neurosci..

[30] Marks KL (2018). Auditory-somatosensory bimodal stimulation desynchronizes brain circuitry to reduce tinnitus in guinea pigs and humans. Sci. Transl. Med..

[31] Salonia A (2012). Is erectile dysfunction a reliable proxy of general male health status? The case for the International Index of Erectile Function-Erectile Function domain. J. Sex. Med..

[32] Gandaglia G (2014). A systematic review of the association between erectile dysfunction and cardiovascular disease. Eur. Urol..

[33] Feldman HA (2000). Erectile dysfunction and coronary risk factors: Prospective results from the Massachusetts male aging study. Prev. Med..

[34] Fung MM, Bettencourt R, Barrett-Connor E (2004). Heart disease risk factors predict erectile dysfunction 25 years later: The Rancho Bernardo Study. J. Am. Coll. Cardiol..

[35] Trune DR, Nguyen-Huynh A (2012). Vascular pathophysiology in hearing disorders. Semin. Hear..

[36] Yıldırım, N. Hearing impairment in vascular disorders. *journalagent.com* (2012).

[37] Chen K-F, Liang S-J, Lin C-L, Liao W-C, Kao C-H (2016). Sleep disorders increase risk of subsequent erectile dysfunction in individuals without sleep apnea: A nationwide population-base cohort study. Sleep Med..

[38] Nguyen HMT, Gabrielson AT, Hellstrom WJG (2017). Erectile dysfunction in young men—a review of the prevalence and risk factors. Sex. Med. Rev..

[39] Retzler K (2019). Erectile dysfunction: A review of comprehensive treatment options for optimal outcome. J. Restor. Med..

[40] Ho CCK, Singam P, Hong GE, Zainuddin ZM (2011). Male sexual dysfunction in Asia. Asian J. Androl..

[41] Ng CJ, Tan HM, Low WY (2008). What do Asian men consider as important masculinity attributes? Findings from the Asian Men’s attitudes to life events and sexuality (MALES) study. J. Mens Health.

